# Extraction of Acoustic Features via Empirical Wavelet Transform to Determine Stenosis Degree of the Left Anterior Descending Artery Based on the Diastolic Heart Sounds of 75 Participants

**DOI:** 10.1111/anec.70195

**Published:** 2026-05-02

**Authors:** Haixia Li, Yafang Zhang, Guofeng Ren, Yun Tian, Yan Chai, Xiaoyan Wang

**Affiliations:** ^1^ Department of Electronics Xinzhou Normal University Xinzhou City Shanxi Province China; ^2^ Department of Cardiology Xinzhou People's Hospital Xinzhou City Shanxi Province China

**Keywords:** acoustic features, coronary artery disease, diastolic murmurs, left anterior descending artery, spectrum energy, stenosis degree

## Abstract

**Objectives:**

This study aimed to develop a method for extracting acoustic features to assess left anterior descending artery (LAD) stenosis severity.

**Methods:**

Heart sound data were collected from 75 participants (10 diastoles per participant) using a high‐signal‐to‐noise ratio micro‐electro‐mechanical systems stethoscope. The diastolic signals were preprocessed, and empirical wavelet transform was applied to decompose their power spectra into three modes (0–150, 150–500, and > 500 Hz). The spectral energies (e(1), e(2), e(3)) of these modes were analyzed, and support vector machine (SVM) and extreme gradient boosting (XGBoost) machine learning algorithms were used to classify LAD stenosis into mild (< 50%), moderate (50%–75%), and severe (> 75%).

**Results:**

Spectral energies e(2) and e(3) significantly increased with stenosis severity, and XGBoost outperformed SVM, achieving a test accuracy of 0.8133 and areas under the curve of 0.9358, 0.9644, and 0.9580 for mild, moderate, and severe stenosis, respectively.

**Conclusion:**

Empirical wavelet transform‐extracted spectral energies of e(2) and e(3), combined with XGBoost, effectively determine LAD stenosis degree, offering a non‐invasive screening tool.

## Graphical abstract

This study proposes a non‐invasive approach for coronary artery disease (CAD) screening by analyzing diastolic heart sounds using an Empirical Wavelet Transform (EWT). Spectral energies e(2) and e(3) were identified as sensitive indicators of left anterior descending artery (LAD) stenosis severity. An XGBoost model achieved high diagnostic accuracy (AUC = 0.96), effectively classifying mild, moderate, and severe stenosis. The method offers a low‐cost, rapid tool to complement coronary angiography in clinical practice.

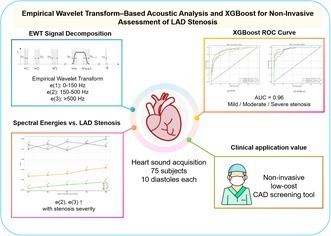

## 1 Introduction

Despite improvements in our material living standards, coronary artery disease (CAD) has become the number one killer of humans globally, with mortality from the condition increasing at an alarming rate (Khare et al. 2023; Nasarian et al. 2020). Early detection and treatment are effective ways to reduce the mortality rate of CAD (Sreenivasan et al. 2024; Pathak et al. 2020).

Currently, coronary angiography is the gold standard for identifying the degree of coronary artery stenosis. However, this method is invasive and requires complex surgical procedures. Thus, there is an urgent need to develop a non‐invasive, convenient, and accurate method for the general screening of CAD and stenosis degree (Ainiwaer et al. 2023).

As a non‐invasive examination method, phonocardiography (PCG) has been widely used to assist in the diagnosis of CAD (Zhao et al. 2025). Previous studies have shown that when the coronary artery is 25% blocked, specific diastolic murmurs appear (Li et al. 2025; Winther et al. 2021). Recently, diastolic turbulent murmurs have been applied to distinguish CAD from non‐CAD (Winther et al. 2021; Rivas‐Navarrete et al. 2025; Sood et al. 2025; Winther et al. 2018; Aiba et al. 2025; Zhao et al. 2023). However, these studies could only distinguish between CAD and non‐CAD and could not determine the blockage degree of the coronary artery. An accurate determination of the degree of coronary artery blockage is essential for doctors to provide appropriate treatment plans that control the development of CAD and thus reduce its mortality rate.

Existing studies that focus on stenosis degree assessment have limitations. For example, Andreini et al. identified three types of patients—those without stenosis, those with coronary artery stenosis of < 50% and those with coronary artery stenosis of > 50%—using coronary computed tomography (CT) image features and a deep convolutional neural network, but the classification effect in this study was not sufficient (Andreini et al. 2018). Alam et al. used an artificial intelligence algorithm (the Naïve Bayes algorithm) to automatically analyze features on coronary CT angiography images from patients in the same standard category, and the algorithm demonstrated better predictive accuracy and sensitivity than linear support vector regression (Alam et al. 2019). Yildirim et al. compared the effectiveness of V3 and V2 scoring methods in assessing the risk stratification of CAD and distinguished CAD from non‐CAD based on heart sound signals combined with clinical symptoms, with a classification sensitivity and specificity of 80.4% and 53%, respectively (Yildirim et al. 2025). Akbar H et al. collected the electrocardiogram (ECG) results and echocardiographic characteristics of patients with CAD and used the Gensini score to classify the high‐ and low‐risk degrees of patients with CAD (Akbar and Mountfort 2024). Acharya et al. analyzed characteristics on coronary artery CT images from patients in three categories—those with calcified plates, those with non‐calcified plates and those with normal coronary arteries—using the Gabor transform combined with a probabilistic neural network, and it achieved an accuracy of 89.09% (Ueyama et al. 2025).

Our own previous study found differences in diastolic murmurs between valvular disease and CAD. Diastolic murmurs in CAD are mainly concentrated above 250 Hz, whereas those in valvular disease contain more energy below 250 Hz (Aiba et al. 2025). Dragomir found that the typical PCG signatures in CAD are a weakened spectral energy below 150 Hz and an increased spectral energy above 150 Hz (Ainiwaer et al. 2023). In the current study, we found that as the stenosis degree of the left anterior descending artery (LAD) increased, diastolic spectral energy increased accordingly. This motivated us to use diastolic spectral energy to identify the stenosis degree of the LAD. Using empirical wavelet transform (EWT), the diastoles of CAD were segmented into three frequency bands: 0–150, 150–500, and > 500 Hz (Li et al. 2019), and the spectral energies of these three frequency bands were obtained as the features that would identify the stenosis degree of the LAD. The band boundaries were determined based on the prior literature (Ainiwaer et al. 2023; Aiba et al. 2025) and a systematic sensitivity analysis (alternative boundaries: 0–120, 120–450, and > 450 Hz; and 0–180, 180–550, and > 550 Hz) to ensure robustness, with the final 0–150/150–500/> 500‐Hz setting achieving the best trade‐off between classification performance and clinical interpretability. The focus of this study is to analyze and compare the features of different stenosis degrees of the LAD and to determine the sensitive features that can be used to identify this stenosis degree. Two machine learning algorithms (support vector machine [SVM] and extreme gradient boosting [XGBoost]) were used to complete LAD stenosis degree classification.

## 2 Method

### 2.1 Data Collection

The heart sound data in this study were collected using a self‐made microelectromechanical system (MEMS) electronic stethoscope with a high signal‐to‐noise ratio (SNR) (Li et al. 2019). The stethoscope was placed at the fourth intercostal space on the left sternal border (i.e., the standard auscultation landmark for LAD‐related heart sounds) closest to the LAD. This study was conducted in accordance with the Declaration of Helsinki and approved by the Ethics Committee of the Second Affiliated Hospital of Shanxi Medical University (approval number: 2021YX069). All participants provided their written informed consent prior to participation.

Coronary blood flow is greatly affected by aortic pressure. The magnitudes of the pressure gradient and of coronary blood flow depend on the capacity of the heart to contract. The features of coronary blood flow through the coronary arteries are not identical in each cardiac cycle. For example, coronary blood flow through the left coronary artery is at its highest in the early diastolic period, whereas coronary blood flow through the right coronary artery is at its highest in the peak systolic period. Therefore, the intensity and distribution of energy in each diastole of the same patient with CAD can still vary.

The inclusion criteria for participants were as follows: (1) patients who had undergone a coronary angiography at the study hospital and had a clear LAD stenosis degree confirmed by angiography; (2) patients with a normal ECG, no history of cardiovascular disease, and no abnormal heart sounds detected through physical examination; and (3) patients aged 18–80 years who were able to cooperate with heart sound data collection.

The exclusion criteria were as follows: (1) patients with conditions such as severe valvular heart disease, congenital heart disease, cardiomyopathy or acute myocardial infarction (100% blockage) that could interfere with heart sound signals; (2) patients with coronary artery calcification (75% blockage) that would affect stenosis assessment; (3) patients with an arrhythmia (e.g., atrial fibrillation) or severe pulmonary disease that could distort heart sound signals; and (4) patients with skin diseases or chest deformities that would prevent the proper placement of the stethoscope.

A total of 75 participants were selected for this study, and 10 diastoles were extracted from each participant. The stenosis degree of the LAD of each patient was determined by coronary angiography at the study hospital. The details of the classified collection of heart sound data are shown in Table 1. Additional baseline characteristics (including hypertension and diabetes histories and smoking status) are provided in Table S1. To account for potential age and sex confounding, a stratified analysis by age (≤ 60 and > 60 years) and sex was performed, with the results confirming that e(2) and e(3) remained significantly associated with the LAD stenosis degree after stratification (*p* < 0.05 for all subgroups).

**TABLE 1:** Information on data collection.

	Normal	Blockage 30%	Blockage 40%–50%	Blockage 50%–60%	Blockage 70%–75%	Blockage 85%	Blockage 90%–95%	ALL
Number of subjects	10	10	10	15	10	10	10	75
Number of diastole	100	100	100	150	100	100	100	750
Male	5	6	7	7	6	8	7	46
Female	5	4	3	8	4	2	3	29
Age	42 ±10.3	57 ±9.9	60.1 ±9.8	58 ±8.7	**65.3** ±10.3	71 ±10.8	70 ±10.5	60.4 ±8.3

### 2.2 Preprocessing

Because this study focuses on the extraction of diastole features, it was necessary to preprocess (i.e., resample, denoise, normalize and segment) the collected heart sound signals before accurately locating diastoles. The sampling frequency of all heart sounds after resampling was 2000 Hz. Normalization ensured that the amplitude variation range of all heart sound data was [−1, +1].

Because the self‐made MEMS electronic stethoscope featured a high SNR, a quiet surrounding environment was maintained during data collection, and the stethoscope was placed directly on the fourth intercostal space of the left margin of the sternal bone as the patient lay supine, rendering noise reduction in heart sound capture relatively simple. The wavelet threshold denoising algorithm was employed for noise removal, and a Sym3 wavelet basis and three‐layer decomposition were uniformly adopted. The threshold function is defined as follows (Zhou et al. 2023):

(1) $$\beta_{j}={\;\sigma }_{j}^{\text{Noise}}\sqrt{2\log\left(N_{j}\right)},$$

where $N_{j}$ denotes the length of the signal at the decomposition scale, or layer *j*, and $\sigma_{j}^{\text{Noise}}$ denotes the noise variance of layer *j*, which can be calculated using Equation (2):

(2) $$\sigma_{j}^{\text{Noise}}=\frac{\text{median}\left(\left|CD_{j}\right|\right)}{0.6745},$$

where ${\mathrm{CD}}_{j}$ denotes the detail component of the wavelet decomposition in layer *j*.

**FIGURE 1:**
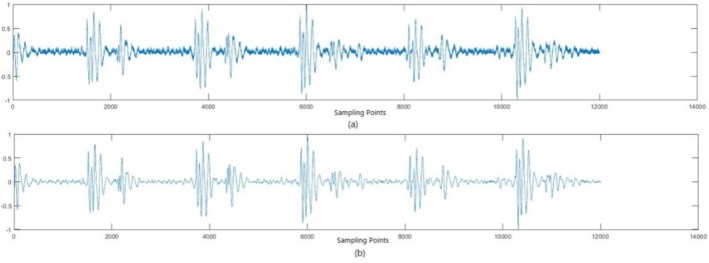
Comparison of a heart sound signal before and after noise removal: (a) before noise removal and (b) after noise removal.

After resampling and normalization, a comparison of the heart sound signal before and after noise removal was conducted. This is shown in Figure 1.

The preprocessing steps before model training were as follows: After extracting spectral energies e(1), e(2) and e(3) and ratios P1 and P2, these features were standardized to have a mean and unit variance of zero. No feature selection was performed, and all extracted features were input into classifiers.

### 2.3 Feature Extraction

**FIGURE 2:**
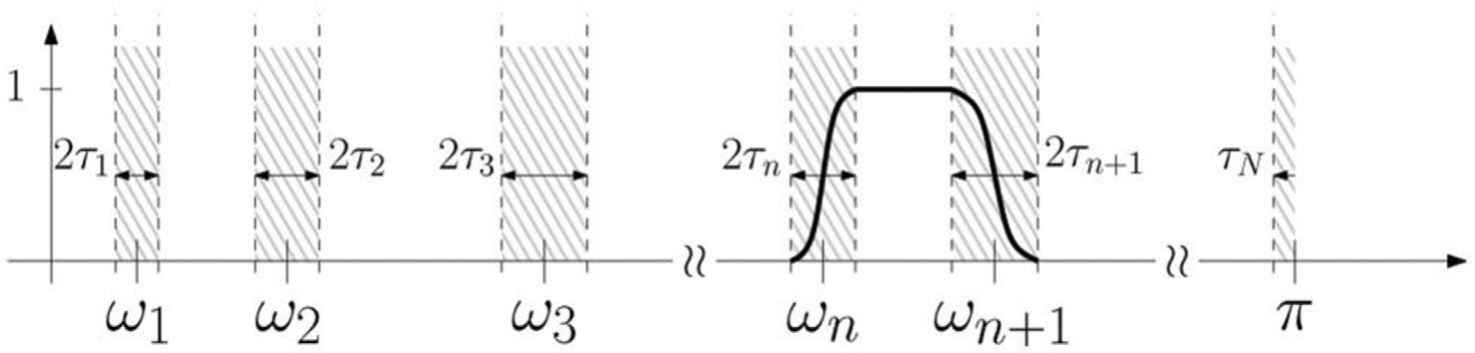
Segmentation of the Fourier spectrum.

Empirical wavelet transform was used to divide the frequency spectrum of the extracted diastolic heart sounds. The EWT algorithm was first proposed by Viswanathan in 2023 (Viswanathan and Palanisamy 2023). Owing to the advantages of the empirical mode decomposition (EMD) algorithm, the EWT algorithm can address the limitations of EMD in producing errors and false modes. The EWT algorithm divides the Fourier spectrum of a signal by constructing a series of bandpass filters within the frequency domain. The determination of filter support depends on the spectral information of the signal to be analyzed. The signal spectrum range is normalized to [0, *π*], and EWT, which is a series of Meyer's wavelet filter banks, is then constructed on each spectrum segment, as shown in Figure 2.

In terms of EWT parameter settings, the number of decomposition modes was set to three; the Meyer wavelet filter bank parameters were set as follows: transition bandwidth (*τ*) = 0.1*π*; and the *β* function was defined as *β*(*x*) = *x* ^2^(3–2*x*) for *x* ∈ [0, 1], satisfying Equation (5).

Diastole was located based on the envelope of the heart sound signal and R‐wave peaks on the synchronized ECG (if available). An ECG was available for 68 of the 75 participants. For the seven participants without an ECG, diastole was located using only the heart sound envelope and detection of the second heart sound (S2) (see below). The detection of S2 end was performed using a two‐step method as follows: (1) S2 candidates were identified via peak detection on the bandpass‐filtered (200–2000 Hz) heart sound signal; and (2) S2 end was validated by thresholding the signal envelope, where an envelope amplitude of < 10% of the S2 peak amplitude was defined as S2 end. The start of diastole was defined as 100 ms after the end of S2, and the end was set at 128 ms after this start point to form a fixed window from 100 to 228 ms post‐S2. This fixed window was justified based on the prior literature (Ainiwaer et al. 2023) and the observation that early diastole (100–228 ms post‐S2) corresponds to peak coronary blood flow velocity in the LAD, which is most sensitive to stenosis‐induced turbulence. A sensitivity analysis using alternative window lengths (e.g., 100 ms [100–200 ms post‐S2] and 156 ms [100–256 ms post‐S2]) showed minimal changes in classification performance (an area under the curve [AUC] variation of < 0.02), supporting the robustness of the chosen window. This window was chosen to capture the maximum diastolic blood flow while avoiding interference from valve closure sounds (during S2) and systolic components.

The scale function and the empirical wavelet function can be expressed using Equations (3) and (4), respectively:

(3) $$\hat{\phi_{n}}\left(w\right)=\left\{\begin{array}{c} 1,\text{if}\;\left|w\right|\leq w_{n}–\tau_{n}\\ \cos\left[\frac{\pi }{2}\beta \left(\frac{1}{2\tau_{n}}\left(\left|w\right|–w_{n}+\tau_{n}\right)\right)\right],\text{if}\;\\ 0,\text{otherwise} \end{array}\right.w_{n}–\tau_{n}\leq w_{n}+\tau_{n}$$

(4) $$\hat{\;\psi_{n}}\left(w\right)=\left\{\begin{array}{c} 1,\text{if}\;w_{n}+\tau_{n}\leq \left|w\right|\;w_{n+1}–\tau_{n+1}\\ \frac{\cos\left[\frac{\pi }{2}\beta \left(\frac{1}{2\tau_{n+1}}\left(\left|w\right|–w_{n+1}+\tau_{n+1}\right)\right)\right],\text{if}\;w_{n+1}–\tau_{n+1}\leq \left|w\right|\leq w_{n+1}+\tau_{n+1}}{\sin\left[\frac{\pi }{2}\beta \left(\frac{1}{2\tau_{n}}\left(\left|w\right|–w_{n}+\tau_{n}\right)\right)\right],\text{if}\;w_{n}–\tau_{n}\leq \left|w\right|\leq w_{n}+\tau_{n}}\\ 0,\text{otherwise} \end{array}\right.,$$

where *β*(*x*) denotes the function satisfying Equation (5) on the interval [0, 1]:

(5) $$\;\beta \left(x\right)=\left\{\begin{array}{c} 0,\text{if}\;x\leq 0\\ \beta \left(x\right)+\beta \left(1–x\right)=1,\forall x\in \left[0,1\right]\\ 1,\text{if}\;x\geq 1 \end{array}\right.$$

To correctly distinguish CAD from non‐CAD and further distinguish the degree of coronary artery blockage, the 150–500‐Hz frequency band was selected according to previously reported physiological characteristics and commonly used ranges in the relevant literature (Sood et al. 2025). In this way, the diastolic signal was decomposed into three modal signals. The distribution ranges of the three modal frequency spectra were 0–150, 150–500, and >500 Hz, respectively. The spectral energy of the three modes, which is typically used to characterize the transient signal energy in a unit frequency band (unit/Hz), was calculated as the squared algebraic sum of the amplitudes of each frequency in the band.

To extract the maximum diastolic blood flow while avoiding interference from valve sounds, a fixed window function was used for each cardiac cycle, starting from 100 ms after the end of S2 and lasting for 128 ms (Ainiwaer et al. 2023).

The spectral energy corresponding to mode 1 is denoted by e(1), the spectral energy corresponding to mode 2 is denoted by e(2), and the spectral energy corresponding to mode 3 is denoted by e(3). Their calculation formulas can be expressed using Equations ((6), (7), (8)):

(6) $$e\left(1\right)=\int_{k=0\;\mathrm{Hz}}^{150\;\mathrm{Hz}}F_{1}{\left(k\right)}^{2}\mathrm{dk}$$

(7) $$e\left(2\right)=\int_{k=150\;\mathrm{Hz}}^{500\;\mathrm{Hz}}F_{2}{\left(k\right)}^{2}\mathrm{dk}$$

(8) $$e\left(3\right)=\int_{k=500\;\mathrm{Hz}}^{2,000\;\mathrm{Hz}}F_{3}{\left(k\right)}^{2}\mathrm{dk},$$

where $F_{1}\left(k\right)$ denotes the Fourier transform of mode 1, $F_{2}\left(k\right)$ denotes the Fourier transform of mode 2 and $F_{3}\left(k\right)$ denotes the Fourier transform of mode 3.

### 2.4 Statistical Analysis

For a comparative analysis of features, differences in spectral energies e(1), e(2) and e(3) and derived ratios P1 and P2 between groups (participants with CAD vs. those without, pre‐ vs. post‐stenting and different stenosis degrees) were tested using the Mann–Whitney U test (for two independent groups) and the Kruskal–Wallis *H* test (for multiple independent groups), as the data did not follow a normal distribution (Shapiro–Wilk test, *p* < 0.05). A *p*‐value < 0.05 was considered statistically significant and indicative of meaningful differences between groups.

In the model performance evaluation, for the classification tasks (mild, moderate, or severe stenosis), key metrics, including accuracy, precision, recall, specificity, and F1 score, were calculated based on confusion matrices. Receiver operating characteristic (ROC) curves were plotted for each stenosis category (one‐vs‐rest strategy), and AUC was computed to quantify each model's discriminative ability. A statistical comparison of AUC values between SVM and XGBoost was performed using the DeLong test to determine significant differences in model performance (*p* < 0.05 was considered significant).

The sample size (75 participants, 750 diastolic segments) was determined based on a preliminary power analysis. Assuming a medium effect size (Cohen's *d* = 0.5) and a significance level of *α* = 0.05, a minimum of 60 participants was required to achieve 80% statistical power for detecting differences in spectral energy between stenosis groups (calculated using G*Power 3.1 Franz Faul, Universität Kiel, Kiel, Germany). The final sample size exceeded this threshold to account for potential data loss during preprocessing.

### 2.5 Model Configurations and Tuning

The SVM model uses a radial basis function kernel. For SVM configuration, hyperparameters were tuned via five‐fold cross‐validation on the training set with a range of 0.1–10 for the regularization parameter *C* and a range of 0.001–0.1 for the kernel coefficient γ. The optimal parameters were *C* = 1.0 and *γ* = 0.01.

The XGBoost model includes the following hyperparameters: number of estimators (100–500), learning rate (0.01–0.1), maximum tree depth (3–7), and subsample ratio (0.7–1.0). For XGBoost configuration, tuning was performed via five‐fold cross‐validation on the training set using grid search. The optimal parameters were as follows: number of estimators = 300, learning rate = 0.05, maximum tree depth = 5, and subsample ratio = 0.8.

A fivefold stratified cross‐validation scheme was used during model tuning to preserve the class distribution. Final model performance was evaluated on an independent test set (using a participant‐wise split) to avoid overfitting.

### 2.6 Schematic Pipeline

A schematic diagram of the complete workflow is provided in Figure S1, including all of the following steps: data collection → preprocessing (resampling, denoising, and normalization) → S2 detection and diastole segmentation → EWT decomposition → feature extraction (spectral energies e(1), e(2), and e(3) and ratios P1 and P2) → feature standardization → model training (SVM or XGBoost, with cross‐validation tuning) → performance evaluation.

## 3 Results

To illustrate the relationship between LAD stenosis degree and extracted features e(1), e(2), and e(3), a comparative analysis was conducted. First, the differences in features between patients with CAD and those without were studied. Subsequently, the differences in features between patients with CAD before and after stenting were studied. Finally, the change rule of the features of CAD with different stenosis degrees of the LAD was studied.

### 3.1 Comparison of Spectral Energies Between Participants With and Without Coronary Artery Disease

**FIGURE 3:**
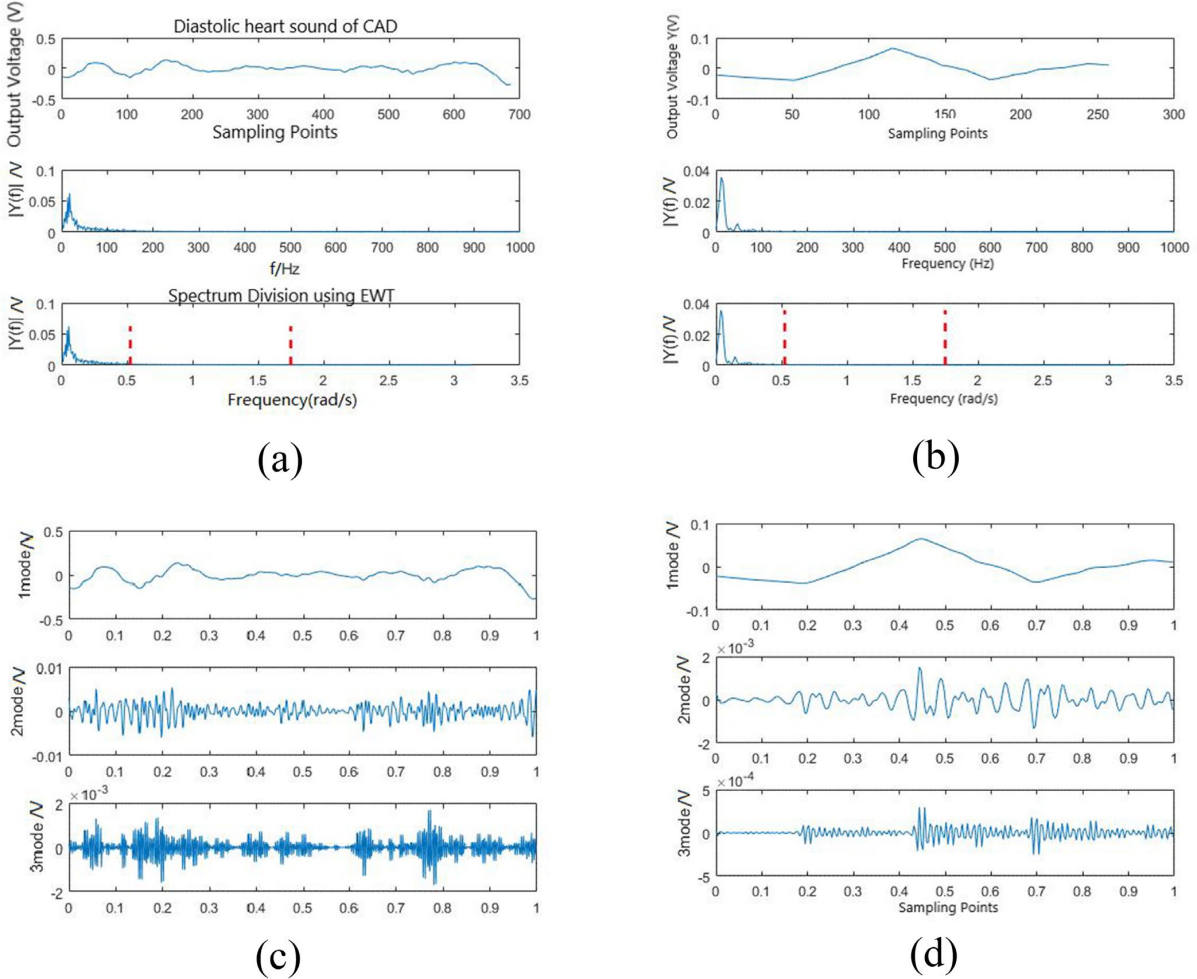
(a) Diastolic spectrum division of CAD. (b) Diastolic spectrum division of a normal subject. (c) Three modal signals of CAD. (d) Three modal signals of a normal subject.

The diastolic signals of the participants with CAD and those without were extracted, and their Fourier spectra were divided by red dotted lines, as shown in Figure 3a,b. It can be clearly observed in Figure 3c,d that more murmurs appeared in the three modal signals of the participants with CAD than in those without CAD.

**FIGURE 4:**
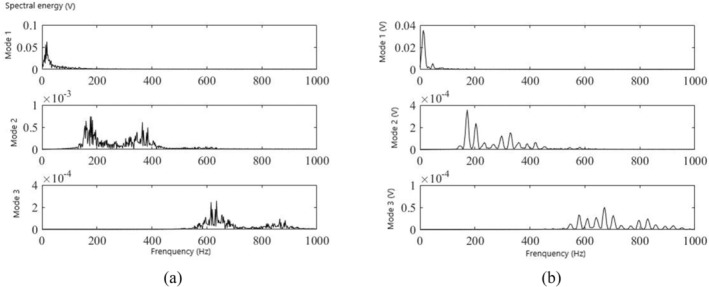
(a) Spectrum of diastolic modes of CAD. (b) Spectrum of diastolic modes of a normal subject.

To observe more clearly the frequency domain features of each modal signal, a Fourier transform was further performed on the three modal signals to obtain the corresponding spectral energy of the three modes, and it can be clearly observed from Figure 4 that there were more spectral energies in the second and third modes in the participants with CAD than in those without.

The features of the 10 diastoles in the patients with CAD with 30% stenosis of the LAD and those in the patients without CAD were calculated, as shown in Table 2. It can be clearly observed from Table 2 that there were significant differences in these features between patients with CAD and those without. The mean value of e(2) in the patients with CAD was 10^−6^, whereas it was 10^−8^ in the patients without. The results suggest that e(1) decreases, but e(2) and e(3) increase in patients with CAD compared with those without. Therefore, two new features—P1 and P2—were added to Table 1. The calculation method for these two features is presented in Equation (9):

(9) $$P_{1}=\frac{e\left(2\right)}{e\left(1\right)},P_{2}=\frac{e\left(3\right)}{e\left(1\right)}$$

**TABLE 2:** Comparison of diastolic features between CAD and normal subjects.

No.	Features of CAD					Features of a normal subject				
	e(1) V^2^*s	e(2) V^2^*s	e(3) V^2^*s	P1	P2	e(1) V^2^*s	e(2) V^2^*s	e(3) V^2^*s	P1	P2
1	0.0015	2.8e−6	1.7e−7	0.0018	1.1e−4	3.4e−4	2.2e−8	1.8e−10	6.4e−5	5.4e−7
2	3.8e−4	1.1e−6	8.9e−8	0.0028	2.3e−4	3.8e−4	3.4e−8	3.4e−10	8.8e−5	8.8e−7
3	7.7e−4	1.9e−6	1.5e−7	0.0025	2.0e−4	9.8e−4	1.6e−8	6.9e−11	1.6e−5	7.0e−8
4	0.0015	1.9e−6	2.4e−7	0.0013	1.6e−4	0.0014	2.1e−8	1.8e−10	1.5e−5	1.3e−7
5	6.2e−4	1.5e−6	1.0e−7	0.0024	1.7e−4	0.0017	1.0e−8	7.8e−11	6.0e−6	4.5e−8
6	4.0e−4	1.1e−6	8.4e−8	0.0029	2.1e−4	0.0013	1.6e−8	1.7e−10	1.2e−5	1.3e−7
Mean	8.6e−4	1.7e−6	1.4e−7	0.0023	1.8e−4	0.0010	2.0e−8	1.7e−10	3.4e−5	3.0e−7

**FIGURE 5:**
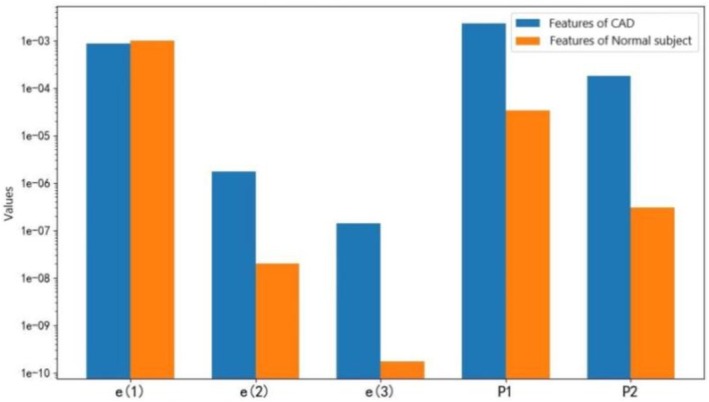
Comparative differences in diastolic features between CAD and normal subjects.

To further intuitively show the differences in features between the patients with CAD and those without, the mean values of the extracted features (e(1), e(2), e(3), P1 and P2) of the 10 diastoles from each patient with CAD with 30% stenosis of the LAD and those of participants without CAD were compared in the form of a bar chart, as depicted in Figure 5. The results of the comparison are consistent with the statistical results presented in Table 2. The mean values of e(2) and e(3) were significantly higher, and e(1) was slightly lower in the patients with CAD than in those without. Additionally, the mean values of P1 and P2 in the patients with CAD were significantly higher than those in the patients without CAD.

### 3.2 Comparison of Diastolic Features of Coronary Artery Disease Before and After Stenting

Understanding how the proposed features of CAD change before and after stent implantation is essential for determining the boundary values of these features in patients with and without CAD and preparing for the classification of the degree of coronary artery stenosis. Therefore, this subsection focuses on the differences in diastolic features before and after stent implantation in patients with a stenosis degree > 75%. It should be noted that, due to the small sample size (three cases) and potential confounding factors (e.g., post‐intervention hemodynamic changes and medication adjustments), this analysis is exploratory.

**FIGURE 6:**
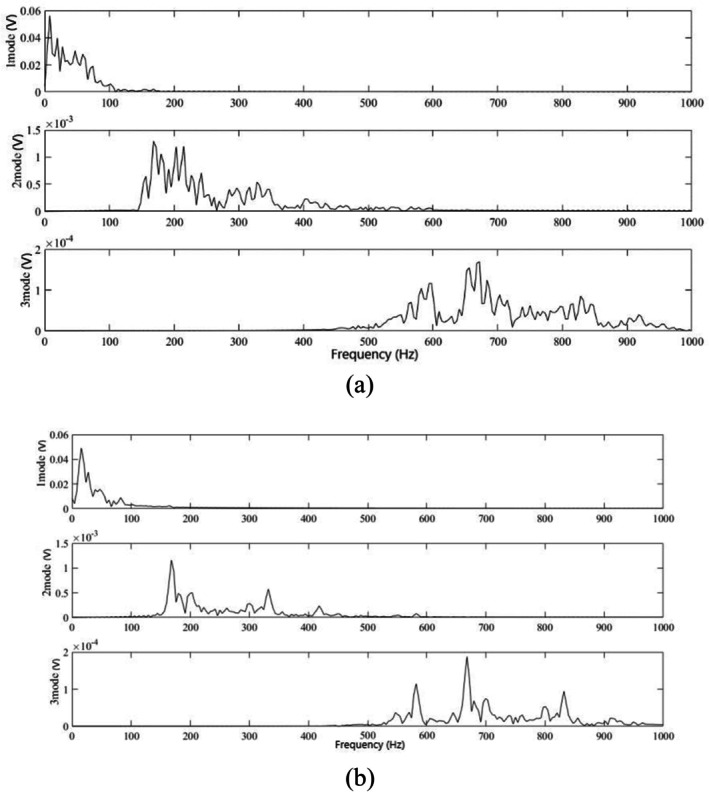
Modal spectrum of three modes: (a) before stenting and (b) after stenting.

To illustrate how modal spectral energy changes before and after stent implantation, a case study is considered as an example. The patient, a 76‐year‐old woman, underwent a coronary angiography, which showed that the proximal segment of the anterior descending branch of the left coronary artery was 95% blocked. A stent was placed in the blockage location, and the heart sounds of this patient were collected on the second day after stent implantation. The diastolic spectra of the three modes in this patient before and after stenting are shown in Figure 6, and it can be clearly observed that the spectral energies in the second and third modes were significantly reduced after stent implantation.

**FIGURE 7:**
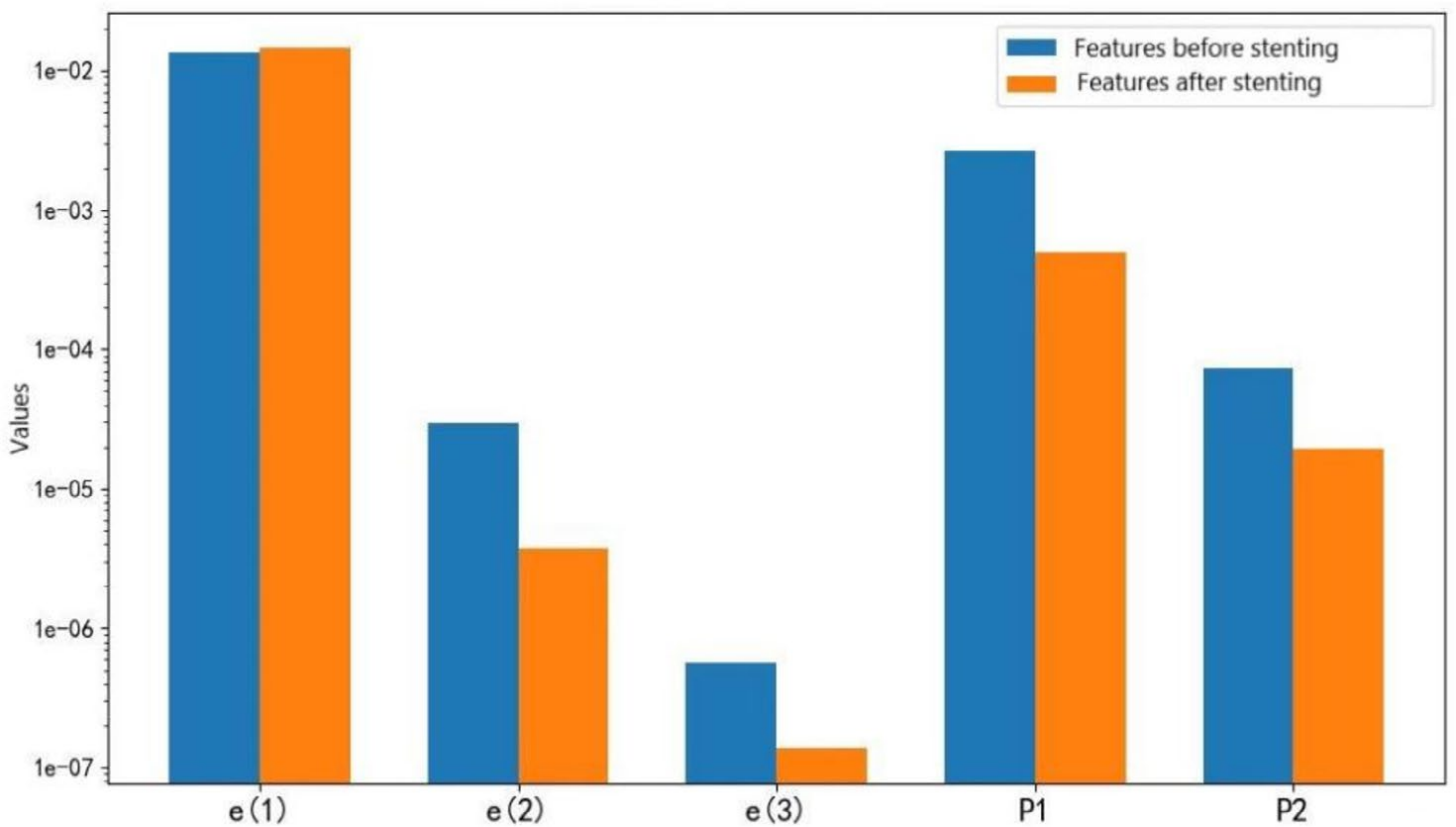
Features before and after the stenting of CAD.

The features of the 10 diastoles in three patients with coronary artery stenosis > 75%—including e(1), e(2), e(3), P1 and P2, as well as their mean values—were collected before and after surgery. To more intuitively observe the comparative changes in these features before and after stent implantation in patients with CAD, the mean value of each feature in the three patients was used to draw bar graphs, as shown in Figure 7. Compared with the values before stent implantation, e(2), e(3), P1 and P2 obviously decreased after stent implantation, and e(1) increased slightly.

### 3.3 Change Rule of Diastolic Features of Coronary Artery Disease With Different Degrees of Stenosis of the LAD

To compare the values of the proposed features of CAD with different stenosis degrees of the LAD, the features of diastoles with LAD stenoses of 30%, 40%, 50%, 75% and 95% were collected, including e(1), e(2), e(3) and P1.

**FIGURE 8:**
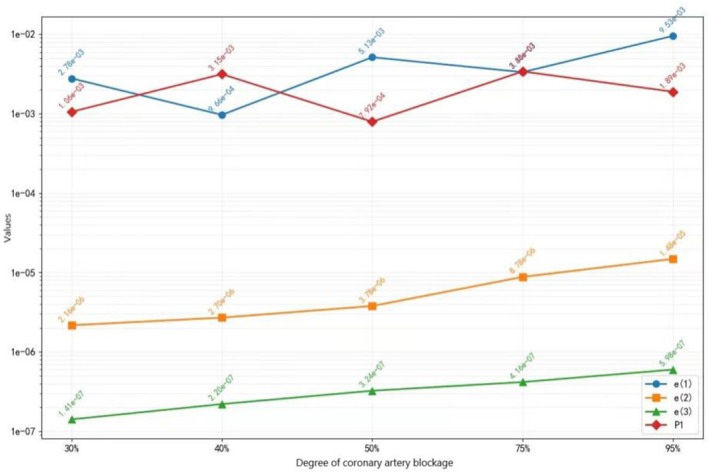
Chang rule of diastolic features with an increase in coronary artery stenosis degree.

Based on the mean values of the features with various stenosis degrees of the LAD, the curve of the change in features according to increasing stenosis degree in the LAD is depicted in Figure 8, and from this figure, the following conclusions can be drawn: (1) with an increase in the degree of stenosis of the LAD, e(2) and e(3) increase; (2) when the stenosis degree is approximately 30%, the value of e(2) reaches 10^−6^; and (3) e(1) and P1 do not increase with an increase in stenosis degree and should be eliminated when identifying the stenosis degree of the LAD.

### 3.4 Classification Performance

To date, the methods of distinguishing CAD from non‐CAD based on diastolic murmurs can generally be divided into two categories. One is machine learning, which involves first extracting the features of diastolic murmurs and then inputting them into various training models. The algorithms commonly used for extracting features include spectral estimation methods (Larsen et al. 2021), fast Fourier transform (Ainiwaer et al. 2023), wavelet analysis (Rivas‐Navarrete et al. 2025) and fast‐tracking filters (Ling et al. 2022), among others, and the machine learning algorithms include SVM, XGBoost, and random forest (Li et al. 2021). The other category is deep learning, which includes convolutional and deep convolutional neural networks (Abdar et al. 2021; Lin et al. 2022).

This study primarily focuses on the accuracy and specificity of extracted diastolic features—namely, the second‐ and third‐mode spectral energies, e(2) and e(3)—in identifying the stenosis degree of the LAD using two machine learning algorithms: SVM and XGBoost.

#### 3.4.1 Data for Model Training and Testing

After excluding special cases (e.g., coronary artery calcification with 75% blockage and acute myocardial infarction with 100% blockage), 750 diastolic segments (10 per participant) from 75 participants were included. These data were split using a participant‐wise strategy into 600 segments (from 60 participants) for training and 150 segments (from 15 participants) for testing, with class distribution preserved (mild: 50 segments [5 participants], moderate: 40 segments [4 participants] and severe: 60 segments [6 participants] in the test set). The stenosis degree of the LAD was categorized into three groups based on clinical standards as follows: mild (< 50%), moderate (50%–75%) and severe (> 75%).

#### 3.4.2 Classification Performance of Support Vector Machine and Extreme Gradient Boosting

**FIGURE 9:**
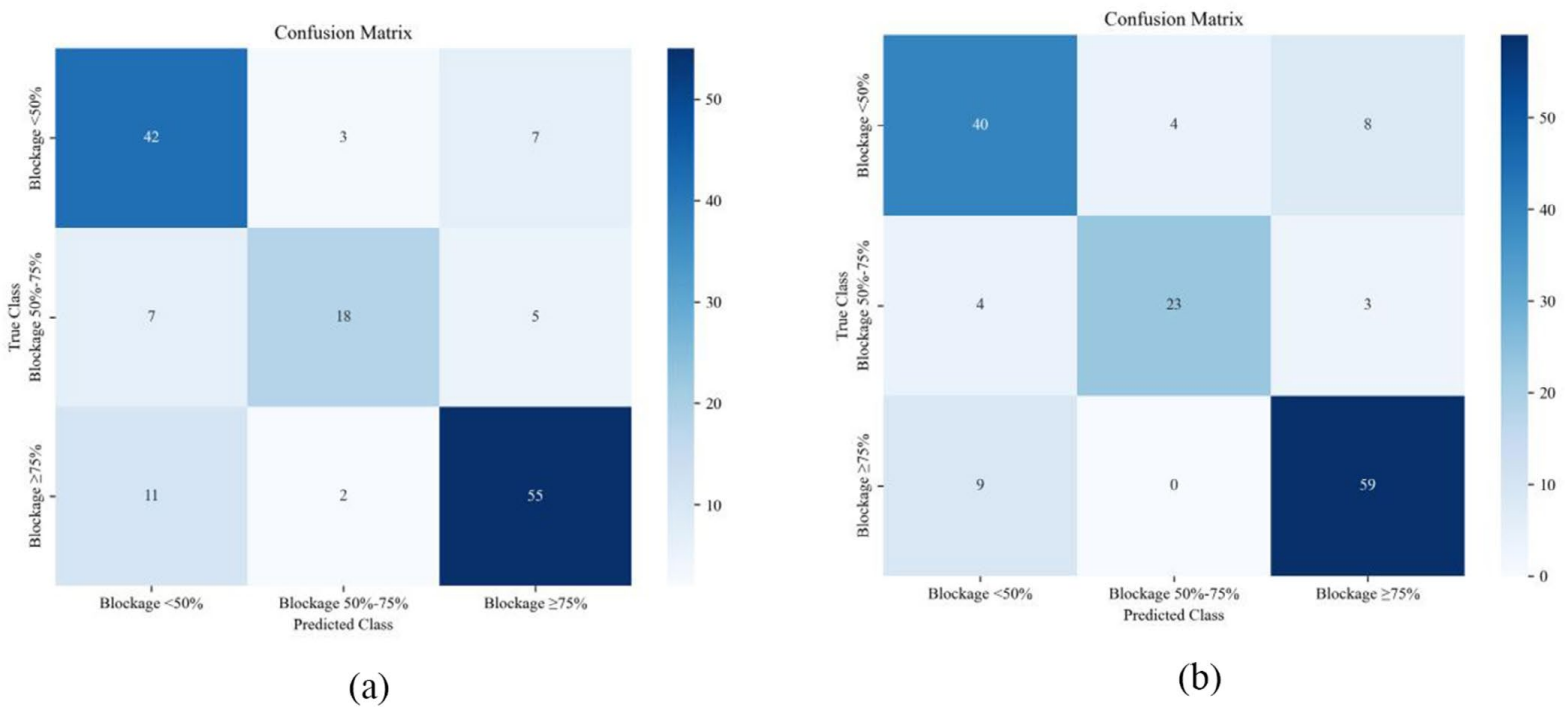
Confusion matrix: (a) SVM and (b) XGBoost.

The performance of the two models was assessed using confusion matrices and ROC curves. The confusion matrices (Figure 9) showed that the test accuracy was 0.7667, the balanced accuracy was 0.7522, and the macro‐F1 score was 0.7489 for the SVM model, whereas the same metrics were 0.8133, 0.8011, and 0.8005, respectively, for the XGBoost model, indicating that XGBoost had a better overall recognition effect.

**FIGURE 10:**
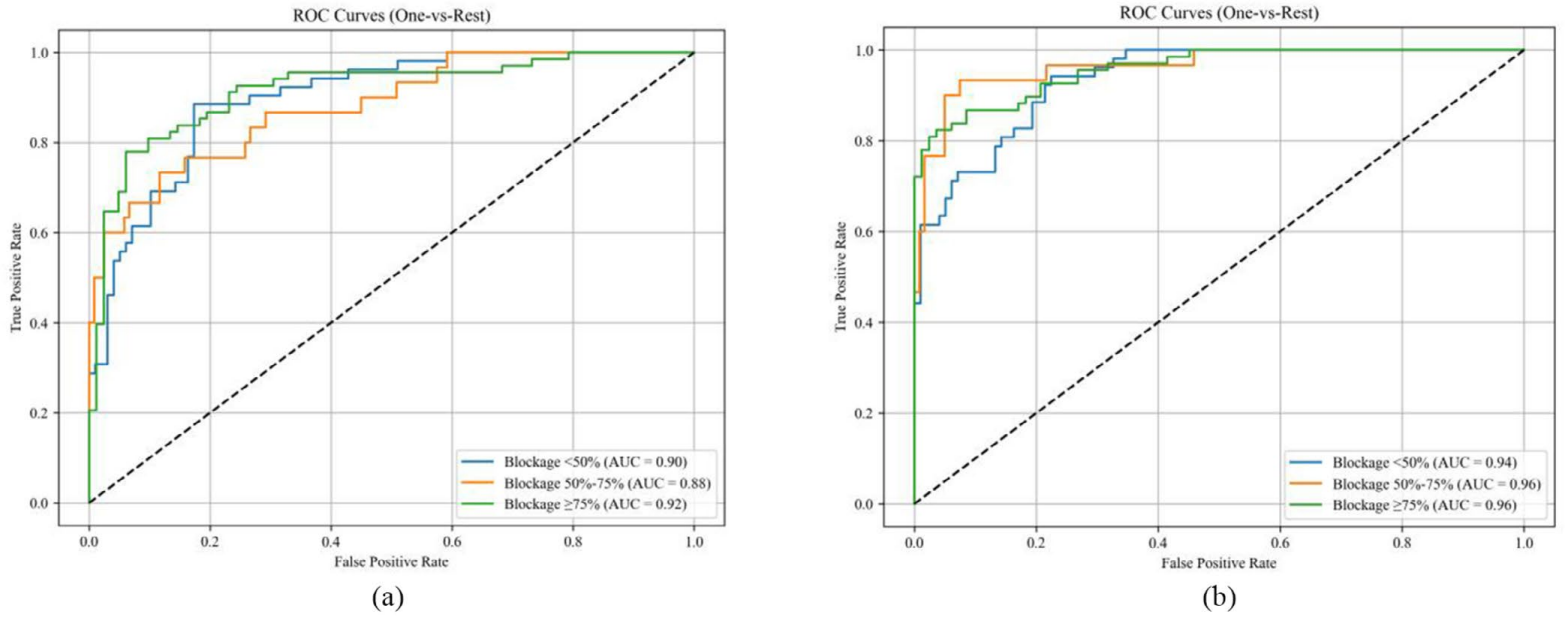
ROC Curves: (a) SVM; (b) XGBoost.

The ROC curves (Figure 10) further demonstrated the discriminative ability of the models. For the SVM model, the AUCs for mild, moderate, and severe stenosis were 0.8993 (95% CI: 0.8412–0.9574), 0.8814 (95% CI: 0.8156–0.9472), and 0.9159 (95% CI: 0.8687–0.9631), respectively, with an average AUC of 0.8989. By contrast, the XGBoost model achieved higher AUCs as follows: 0.9358 (95% CI: 0.8921–0.9795) for mild stenosis, 0.9644 (95% CI: 0.9312–0.9976) for moderate stenosis, and 0.9580 (95% CI: 0.9203–0.9957) for severe stenosis, with an average AUC of 0.9528.

#### 3.4.3 Detailed Classification Metrics

Detailed classification metrics (Table 3) reveal that XGBoost outperformed SVM in most indicators. For mild stenosis, XGBoost showed higher specificity (0.8673 vs. 0.8163) and a higher AUC (0.9358 vs. 0.8993). For moderate stenosis, it achieved higher precision (0.8517 vs. 0.7826) and a higher F1 score (0.8070 vs. 0.6792). For severe stenosis, XGBoost had higher recall (0.8676 vs. 0.8088) and a higher AUC (0.9580 vs. 0.9159). These results collectively indicate that, using extracted acoustic features, the XGBoost model is more effective in classifying LAD stenosis degree.

**TABLE 3:** Classification results.

SVM						XGBoost				
Blockage degree	Precision	Recall	Specificity	F1 score	AUC	Precision	Recall	Specificity	F1 score	AUC
< 50%	0.7000	0.8077	0.8163	0.7500	0.8993	0.7547	0.7692	0.8673	0.7619	0.9358
50%–75%	0.7826	0.6000	0.9583	0.6792	0.8814	0.8517	0.7667	0.9667	0.8070	0.9644
> 75%	0.8209	0.8088	0.8537	0.8148	0.9159	0.8429	0.8676	0.8659	0.8551	0.9580

Based on the confusion matrices and ROC curves, the precision, recall, specificity, F1 score, and AUC of each of the two models were calculated, as shown in Table 3.

## 4 Discussion

This study collected different stenosis degrees of the LAD using our self‐made, high‐SNR MEMS stethoscope. Although the amount of collected data is not large (totalling 75 participants), the categories within this cohort are comprehensive for this study, including 10 participants without CAD and 10 participants with 30% stenosis, 10 participants with 40% stenosis, 15 participants with 50%–60% stenosis, 10 participants with 70%–75% stenosis, 10 participants with 85% stenosis, and 10 participants with 90%–95% stenosis of the LAD. The stenosis locations of all the participants with CAD occurred in the LAD. The 75 data points were selected because the stethoscope was placed close to the LAD. After preprocessing, including resampling, normalization, and denoising, the classical envelope extraction and double‐threshold positioning algorithms were adopted to extract 10 diastoles from each heart sound data set (one per participant). A fixed window function from 100 to 128 ms after the end of S2 was used to extract diastolic heart sounds to obtain the maximum diastolic blood flow while avoiding the influence of valve sounds.

The core of this study lies in the use of the EWT algorithm to extract diastolic modal spectral energies as features for identifying the degree of blockage of the LAD. By segmenting the diastolic heart sound spectrum into three bands (0–150, 150–500, and > 500 Hz) and defining their spectral energies (e(1), e(2), and e(3)) as features, comparative analyses revealed that e(2) and e(3) increased with the severity of LAD stenosis (except in special cases, such as acute myocardial infarction and coronary artery calcification). This finding extends previous research on diastolic murmurs in CAD; for example, Ainiwaer et al. (Ainiwaer et al. 2023) noted an increase in spectral energy above 150 Hz in CAD, and our study further links this energy change to stenosis degree, providing a quantitative basis for severity assessment.

Classification results using SVM and XGBoost show that XGBoost outperformed SVM in identifying mild, moderate and severe stenosis, with a test accuracy of 0.8133 and an average AUC of 0.9528. This superiority may stem from XGBoost's ability to handle non‐linear relationships between spectral energy features and stenosis degree, which aligns with the complex hemodynamic changes underlying coronary stenosis. Compared with imaging‐based methods (e.g., coronary CT angiography (Li et al. 2019; Ling et al. 2022)), our acoustic approach offers a non‐invasive, low‐cost alternative suitable for large‐scale screening to complement existing diagnostic tools.

Some limitations of this study should be acknowledged. First, the sample size was relatively small (75 participants), and all data were collected from a single center, potentially limiting the generalisability of these results. Second, the exclusion of cases with coronary artery calcification and acute myocardial infarction may have reduced the model's robustness in handling complex clinical scenarios. Third, the fixed window for diastolic extraction (100–228 ms post‐S2) may not account for individual variations in cardiac cycle duration, which could introduce noise. Future studies should expand the sample size across multiple centers, include diverse pathological conditions and optimize diastolic segmentation using adaptive windowing to improve feature stability.

In conclusion, this study demonstrates that diastolic spectral energy features (e(2) and e(3)) extracted via EWT can effectively distinguish LAD stenosis degree, with XGBoost providing reliable classification performance. This work contributes to the development of non‐invasive CAD‐severity assessment tools, offering potential value for clinical decision making in resource‐limited settings.

## Author Contributions

Conception and design of the work: Haixia Li. Data collection: Yafang Zhang, Guofeng Ren, Yun Tian, Yan Chai, Xiaoyan Wang. Supervision: Haixia Li. Analysis and interpretation of the data: Yafang Zhang, Guofeng Ren, Yun Tian, Yan Chai, Xiaoyan Wang. Statistical analysis: Haixia Li, Yan Chai, Xiaoyan Wang. Drafting the manuscript: Haixia Li. All authors approved the final manuscript and critical revision of the manuscript.

## Funding

This work was supported in part by the Basic Research Program of Shanxi Province—Youth Project under Grant 202203021222304, in part by Science and Technology Innovation Project of Colleges and Universities in Shanxi Province under Grant J2024L324.

## Ethics Statement

This study was conducted in accordance with the Declaration of Helsinki and approved by the Ethics Committee of the Second Affiliated Hospital of Shanxi Medical University (approval number: 2021YX069). All subjects provided written informed consent prior to participation.

## Consent

The authors have nothing to report.

## Conflicts of Interest

The authors declare no conflicts of interest.

## Supporting information


**Figure S1:** Diagram of the complete workflow.

**Table S1:** Baseline characteristics of the 75 participants stratified by left anterior descending artery.

## Data Availability

The data that support the findings of this study are available from the corresponding author upon reasonable request.
