# Supplementary figures and images for: Extraction of Acoustic Features via Empirical Wavelet Transform to Determine Stenosis Degree of the Left Anterior Descending Artery Based on the Diastolic Heart Sounds of 75 Participants
Source: Ann Noninvasive Electrocardiol. 2026 May 2;31(3):e70195. doi: 10.1111/anec.70195 (PMC13135174; doi:10.1111/anec.70195)

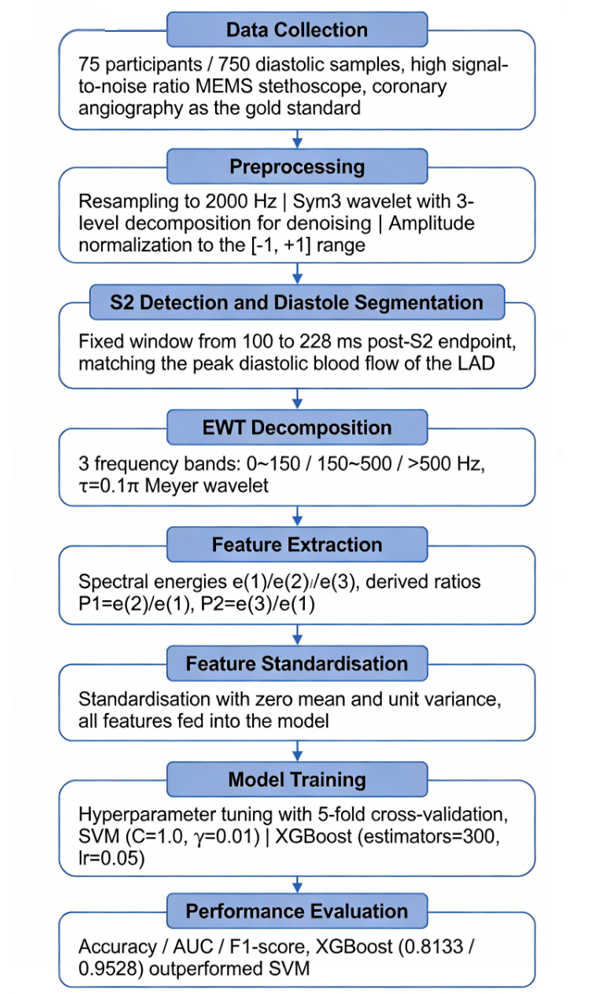

Supplement: Supplementary file 1 — Figure S1: Diagram of the complete workflow. [file ANEC-31-e70195-s001.png]
