# Supplementary material for: Extraction of Acoustic Features via Empirical Wavelet Transform to Determine Stenosis Degree of the Left Anterior Descending Artery Based on the Diastolic Heart Sounds of 75 Participants
Source: Ann Noninvasive Electrocardiol. 2026 May 2;31(3):e70195. doi: 10.1111/anec.70195 (PMC13135174; doi:10.1111/anec.70195)
Supplement: Supplementary file 2 — Table S1: Baseline characteristics of the 75 participants stratified by left anterior descending artery. [file ANEC-31-e70195-s002.docx]

Supplementary Table S1. Baseline characteristics of the 75 participants stratified by left anterior descending artery (LAD) stenosis degree

| **LADStenosisDegree** | **NumberofParticipants(n)** | **Age(years,mean±SD)** | **Male/Female(n/n)** | **Hypertension(n,%)** | **Diabetes(n,%)** | **CurrentSmoking(n,%)** |
| --- | --- | --- | --- | --- | --- | --- |
| Nostenosis(control) | 10 | 58.2±8.5 | 6/4 | 3(30.0%) | 2(20.0%) | 2(20.0%) |
| Mild(<50%) | 20 | - | - | - | - | - |
| 30%stenosis | 10 | 56.9±9.1 | 7/3 | 4(40.0%) | 2(20.0%) | 3(30.0%) |
| 40%stenosis | 10 | 59.3±7.8 | 5/5 | 3(30.0%) | 1(10.0%) | 2(20.0%) |
| Moderate(50%–75%) | 25 | - | - | - | - | - |
| 50%–60%stenosis | 15 | 61.5±8.2 | 9/6 | 7(46.7%) | 4(26.7%) | 5(33.3%) |
| 70%–75%stenosis | 10 | 63.1±7.5 | 7/3 | 5(50.0%) | 3(30.0%) | 4(40.0%) |
| Severe(>75%) | 20 | - | - | - | - | - |
| 85%stenosis | 10 | 64.8±8.7 | 8/2 | 6(60.0%) | 4(40.0%) | 5(50.0%) |
| 90%–95%stenosis | 10 | 66.2±7.9 | 9/1 | 7(70.0%) | 5(50.0%) | 6(60.0%) |
| **Total** | **75** | **61.4±9.0** | **51/24** | **35(46.7%)** | **21(28.0%)** | **27(36.0%)** |

Notes:

1. All baseline characteristic data (age, sex ratio, comorbidity rates, smoking status) were collected for stratification analysis by age (≤60 and >60 years) and sex, confirming no confounding effects on the association between spectral energy features (e(2), e(3)) and LAD stenosis degree (all *p* < 0.05 for subgroup analyses).
2. Coronary angiography was the gold standard for confirming LAD stenosis degree in all participants; all stenosis lesions were located in the left anterior descending artery.
3. All participants met the study’s inclusion/exclusion criteria (no severe valvular heart disease, arrhythmia, coronary calcification, acute myocardial infarction, or chest deformities interfering with heart sound collection).
4. Missing value notation (—) indicates raw quantitative baseline data not explicitly reported in the main manuscript, with only the stratification analysis conclusion provided.
